# Stronger together: Community participation, structural stigma, and depressive symptoms of sexual and gender minority individuals living across 28 European countries: Berk Can Ünsal

**DOI:** 10.1093/eurpub/ckac129.194

**Published:** 2022-10-25

**Authors:** B Ünsal, Z Demetrovics, M Reinhardt

**Affiliations:** Institute of Psychology, ELTE Eötvös Loránd University, Budapest, Hungary

## Abstract

**Background:**

Although previous studies demonstrated that structural stigma (i.e., discriminatory state laws, public policies, and attitudes) predicts adverse mental health outcomes among sexual and gender minority (SGM) populations, less is known how protective factors interact with structural stigma. Thus, we aimed to examine the associations between structural stigma, community participation, and depressive symptoms in a large sample of SGM adults.

**Methods:**

Discriminatory laws, policies, and attitudes affecting SGM people were assessed to measure each country's structural stigma levels (i.e., sexual and gender). Data from the 2019 EU-LGBTI-II-Survey assessing community participation levels and depressive symptoms of sexual minority men (n = 62.825), women (n = 38.912), and gender minority adults (n = 15.801) in 28 European countries were analyzed by using multilevel models.

**Results:**

The results demonstrated that structural stigma was positively, and community participation was negatively associated with depressive symptoms of sexual minority men (β = .147, p < .001; β = -.020, p < .05), women (β = .149, p < .01; β = −.040, p < .01), and gender minority adults (β = .085, p < .05; β = -.088, p < .001), respectively. Unlike sexual minority women and gender minority adults, for sexual minority men, a statistically significant interaction was found (β = .018, p < .05) such that participating to the community predicted lower depressive symptoms only in lower-stigma countries.

**Conclusions:**

The results highlight the need for changes in discriminatory laws, social policies, and negative attitudes that impact depressive symptoms of SGM individuals. Although community participation protects individuals from depression, these findings suggest that sexual minority men in higher-stigma countries benefit less from community participation. Thus, interventions aiming to increase SGM individuals’ community participation should consider structural factors and gender differences.

